# Effect of Steel Slag on the Properties of Alkali-Activated Slag Material: A Comparative Study with Fly Ash

**DOI:** 10.3390/ma17112495

**Published:** 2024-05-22

**Authors:** Fanghui Han, Ziqin Zhu, Hongbo Zhang, Yuchen Li, Ting Fu

**Affiliations:** 1 Beijing Key Laboratory of Urban Underground Space Engineering, School of Civil and Resource Engineering, University of Science and Technology Beijing, Beijing 100083, China; 2 Research Institute of Urbanization and Urban Safety, University of Science and Technology Beijing, Beijing 100083, China; 3 China Construction Science and Technology Group Co., Ltd., Beijing 100070, China

**Keywords:** alkali-activated slag binder, steel slag, mechanical properties, microstructure, drying shrinkage

## Abstract

Slag and fly ash (FA) are mostly used as precursors for the production of alkali-activated materials (AAMs). FA is the waste discharged by power plants, while slag and steel slag (SS) both belong to the iron and steel industry. The effects of SS and FA on the strength, microstructure, and volume stability of alkali-activated slag (AAS) materials with different water glass modulus (Ms) values were comparatively investigated. The results show that adding SS or FA decreases the compressive strength of AAS mortar, and the reduction effect of SS is more obvious at high Ms. SS or FA reduce the non-evaporable water content (W_n_) of AAS paste. However, SS increases the long-term W_n_ of AAS paste at low Ms. The cumulative pore volume and porosity increase after adding SS or FA, especially after adding FA. The hydration products are mainly reticular C-(A)-S-H gels. Adding SS increases the Ca/Si ratio of C-(A)-S-H gel but decreases the Al/Si ratio. However, by mixing FA, the Ca/Si ratio is reduced and the Al/Si ratio is almost unchanged. The incorporation of SS or FA reduces the drying shrinkage of AAS mortar, especially when SS is added. Increasing Ms increases the compressive strength and improves the pore structure, and it significantly increases the drying shrinkage of all samples. This study provides theoretical guidance for the application of steel slag in the alkali-activated slag material.

## 1. Introduction

Portland cement occupies a prominent position among binders. It is widely used in civil construction, water conservancy, national defense, and other fields [1]. China is a major producer and consumer of Portland cement, and its annual production of Portland cement consistently accounts for more than 45% of the global annual production [2]. However, the production of Portland cement expends lots of resources and energy and emits substantial carbon dioxide [3,4]. Alkali-activated materials (AAMs) are formed by the reaction of silicate–aluminate solid raw materials with pozzolanic activity or potential hydraulicity with alkaline activators. AAM is a potential alternative product of Portland cement [5,6]. AAMs have the advantages of abundant raw material sources, high strength [7,8], low production cost, environmental protection, excellent durability [9,10], and good fixation of toxic heavy metal ions [11,12].

Slag is mostly used as a precursor to produce AAMs due to its high activity. The quantity of slag is in short supply owing to the keen demand for construction. The price of is also slag becoming higher. Although the characteristics of alkali-activated slag (AAS) binder have been widely researched, the volume stability of AAS paste is poor [13,14] and strength develops quickly at early age [15]. In addition, the strength of AAS binder is commonly reduced at later age [16]. To realize the purpose of reducing cost, decreasing shrinkage, and making mechanical properties meet the requirements, the partial replacement of slag with FA is mainly used to overcome the shortcomings of AAS binder. The activator of water glass or NaOH has a major effect on the performance of AAS binder. The mechanical properties of alkali-activated slag-fly ash (AASF) concrete can be improved by increasing Ms, the sodium hydroxide concentration, and alkali content [17]. Increasing the water/binder (w/b) ratio decreases the mechanical properties of AASF concrete [18]. The shrinkage of AASF concrete decreases with increasing sodium hydroxide concentration and decreasing Ms [19]. The resistance to fire, chloride ion permeability, freezing, and acidity of AASF concrete are improved with increasing slag content and alkali activator dosage [20,21]. The structure of AASF paste did not change under the attack of Na_2_SO_4_ solution, while the Mg_S_O_4_ solution caused serious erosion on the structure of AASF paste [22]. The pore structure of AAS paste becomes poor after adding FA [23]. The micromechanical characteristics of the interfacial transition zone in AASF concrete are highly dependent on its mix ratio [24].

The activities of SS and FA are lower than that of slag. FA is solid waste from power plants. However, slag and SS can be obtained simultaneously in steel mills. If the alkali-activated slag-steel slag (AASS) binder can obtain similar properties to the AASF binder, the recycling of SS will be realized. Meanwhile, the undersupply of slag and FA for the production of AAMs would also be solved. However, the application of SS to the preparation of AAMs is rarely studied. The hydration products of alkali-activated SS binder were C-S-H gel and Ca(OH)_2_ (CH). The contents of CH and non-evaporable water content (W_n_) decreased with increasing Ms [25]. The alkali activators Na_2_SiO_3_ and Na_2_SO_4_ effectively improved the reactivity of SS, generating more C-S-H gels [26]. Increasing Ms refined the pore structure and improved the strength of the paste [25]. SS plays a better role in the ternary system. Song et al. [27] found that SS and slag significantly improved the mechanical properties of alkali-activated FA binder. Adding SS and slag decreased the brittleness of alkali-activated FA paste. Chen et al. [28] found that the alkali-activated steel slag–fly ash–metakaolin composite binder had relatively low early strength, but the later strength growth was faster. Zhao et al. [29] reported that an alkali-activated composite binder prepared with 15% SS, 60% slag, and 25% FA had the best mechanical properties. However, the mechanical properties declined when the SS content was greater than 15%.

According to the present references, it is clear that the properties of AAS binder have been extensively investigated. AAS binder has excellent mechanical properties, whereas its setting and hardening behaviors are hard to control. In addition, the shrinkage of AAS materials is large, and the strength decreases at a later age. The strength of alkali-activated binder prepared with only SS or FA is lower due to its low activity. However, research on the properties of AASS hardened paste is not yet in depth. The influence mechanism of SS on the microstructure formation of AAS binder is still unclear. Therefore, in this research, 50% slag was replaced with SS in the AAS paste. Water glass was adopted as the activator. The influences of the water glass modulus (Ms) on the mechanical properties, microstructure, and drying shrinkage of AASS pastes were investigated. The effects of SS on the properties of AAS paste were comparatively analyzed and discussed with those of FA. The results of this study further elucidate the properties of the AASS binder and play a certain role in promoting the efficient utilization and recycling of SS.

## 2. Materials and Methods

### 2.1. Raw Materials

**Figure 1:**
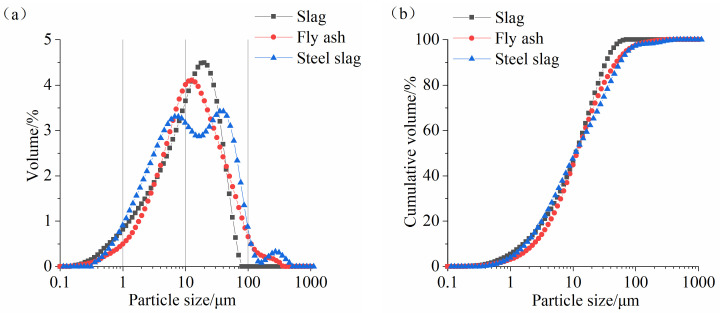
Particle size distribution curves of raw materials. (**a**) Differential curve and (**b**) cumulative volume.

**Figure 2:**
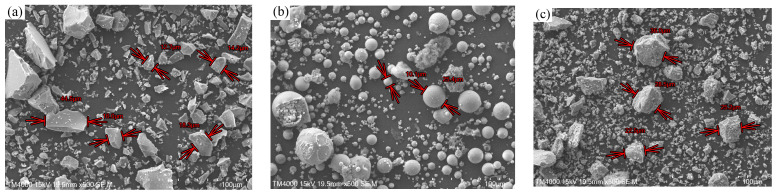
Morphology of the raw materials: (**a**) slag, (**b**) FA and (**c**) SS.

S95 blast furnace slag, Class I FA, and basic oxygen furnace SS were used in this study. S95 GGBS, Class I FA, and basic oxygen furnace SS were produced by Shandong JinTaicheng Construction Technology Co., Ltd., Linyi, China. The chemical compositions of the three raw materials are presented in Table 1. The main components of slag are SiO_2_, CaO, Al_2_O_3_, and MgO. The major compositions of FA are SiO_2_ and Al_2_O_3_, but the content of CaO is the lowest. The main components of SS are CaO, Fe_2_O_3_, and SiO_2_. For SS, the content of SiO_2_ is lower than that of the other two materials, but the contents of CaO and Fe_2_O_3_ are relatively high. The particle size distribution curves of three materials are given in Figure 1. Slag is slightly finer than FA, and SS has the smallest particle size. Owing to the existence of the RO phase in the SS, large particles (>100 μm) are found. The morphology of the three materials are given in Figure 2. Slag is an irregular particle (Figure 2a). FA is a solid or hollow spherical particle (Figure 2b). There are more fine particles in SS, and the difference between large particles and small particles is large (Figure 2c).

Water glass was used as the activator. The reasons for choosing water glass as alkali activator were as follows: water glass contained strong alkali sodium hydroxide, which was conducive to stimulating the hydration activity of materials such as fly ash. In addition, water glass contained silicon ions, which was conducive to increasing the amount hydration products. The Ms of the water glass was adjusted by adding NaOH, which was an analytical reagent. Sodium silicate solution was produced by Shandong Yousuo Chemical Technology Co., Ltd., Linyi, China. Sodium hydroxide was an analytical reagent produced by Xilong Chemical Co., Ltd., Shantou, China. Laboratory tap water was used for the preparation of samples. A superplasticizer produced by Jiangsu Sobute New Materials Co., Ltd., Nanjing, China was added to guarantee the fluidities of the paste and mortar. The mortar was prepared with ISO standard sand produced by Xiamen ISO Standard Sand Co., Ltd., Xiamen, China.

### 2.2. Mix Proportions

Water glass activators with different Ms values were prepared 24 h in advance before the experiments to release abundant heat during the preparation process. The w/b ratio was 0.4. The dosage of superplasticizer was 1% of the mass of the binder. The mix proportions of the pastes and mortars are shown in Table 2 and Table 3, respectively.

### 2.3. Test Methods

The paste and mortar samples were prepared according to Table 2 and Table 3, respectively. The properties of the paste and mortar specimens were measured after curing to a certain age with standard curing conditions (temperature of 20 ± 1 °C, relative humidity (RH) ≥ 90%).

The flexural strength and compressive strengths of mortars cured for 3 d, 7 d, 28 d, and 90 d were measured. Three and six specimens were tested in the flexure and compressive strength tests, respectively, for everybody type of specimen.

The W_n_ of the pastes were measured by using the heating ignition method. These samples were ground and placed in anhydrous ethanol to terminate the hydration for 24 h at the test ages. Then, the samples were placed in an oven at 60 °C until constant weight. After that, the weighed samples (m_1_) were placed in a muffle furnace, cauterized from 20 °C to 1000 °C for 2 h, and then kept at 1000 °C for 1 h. When the samples were cooled to 20 °C, the samples were weighed (m_2_). The W_n_ was calculated by Equations (1) and (2).

(1) $$W_{n}=\frac{m_{1}-m_{2}}{m_{1}}-\frac{W_{\mathrm{GS},(\mathrm{FA},\mathrm{SS})}}{{1-W}_{\mathrm{GS},(\mathrm{FA},\mathrm{SS})}}$$

(2) $$W_{\mathrm{GS},(\mathrm{FA},\mathrm{SS})}=W_{\mathrm{GS}}\times L_{\mathrm{GS}}+W_{\left(\mathrm{FA},\mathrm{SS}\right)}\times L_{\left(\mathrm{FA},\mathrm{SS}\right)}$$

where W_n_ represents non-evaporable water content (%); m_1_ and m_2_ represent the mass of the sample at 60 °C and at 1000 °C, respectively; W_GS_ and W_(FA,SS)_ are the mass fractions of slag (%) and FA (or SS) (%), respectively; and L_GS_ and L_(FA,SS)_ are the loss on ignitions of slag and FA (or SS), respectively.

The hydration products of the pastes were investigated using a TTR Ⅲ X-ray diffraction instrument at a scanning speed of 8°/min in the 2θ range of 5–70°.

The middle section of the sample was intercepted and cut into pieces, soaked in anhydrous ethanol for 24 h, and then dried at 40 °C for 30 min before inlaying in resin. Then, they were ground and polished with an automatic sample grinder for the BSE test. The microstructure of the paste was observed with a scanning electron microscope in BSE mode. The compositions of the C(N)-(A)-S-H gels in the pastes at 28 d were determined by analyzing 80 microregions of the C(N)-(A)-S-H gels with energy dispersive spectroscopy (EDS). The average Ca/Si and Al/Si ratios of the C(N)-(A)-S-H gels were calculated.

The paste specimens were cured for 90 d and broken into small pieces of approximately 1 cm^3^. The differential pore volume and cumulative pore volume of the samples were determined by using a mercury intrusion porosimetry instrument from Micromeritics instrument (Shanghai) Ltd., Shanghai, China. The characteristics of the pore structure were analyzed.

The drying shrinkage test of mortars was performed. The specimen size was 25 mm × 25 mm × 280 mm. Three specimens were tested for each mixing ratio. After standard curing for 24 ± 2 h, the specimens were put in water for 48 h. The surface water and dirt on the nail head were wiped off, and the initial reading (L_0_) was measured with a specific length meter. Then, the samples were put into a drying shrinkage curing box with curing conditions of 20 ± 3 °C and 50 ± 4% RH. The changes in the drying shrinkage of mortars were measured up to 90 d, and the average drying shrinkage rates were calculated. The drying shrinkage rate was calculated according to Equation (3):

(3) $$S=\frac{L_{0}-L}{250}$$

where S is the drying shrinkage rate (μm/m); L_0_ is the initial measured length of mortar (mm); L is the measured length of mortar at corresponding period (mm); and 250 is the equivalent length of the sample (mm).

## 3. Results and Discussion

### 3.1. Mechanical Properties

**Figure 3:**
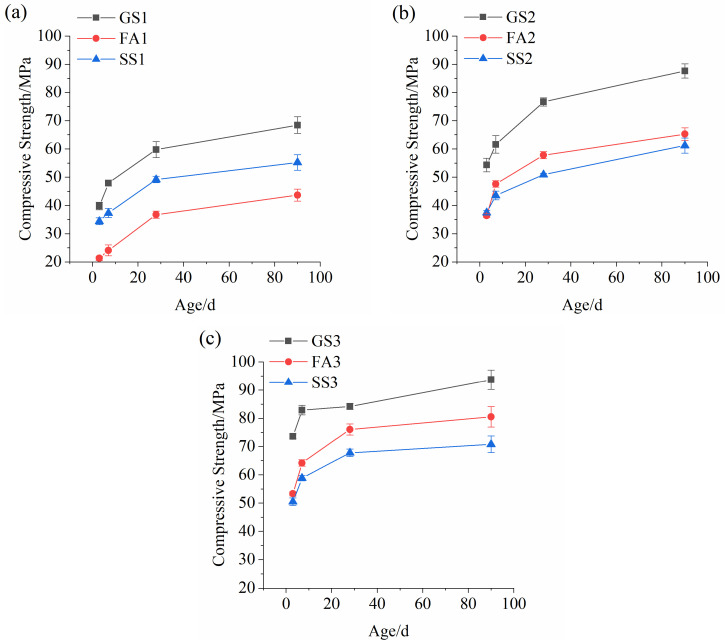
Compressive strengths of AASS and AASF mortars with different Ms values: (**a**) 0.5 Ms, (**b**) 1.0 Ms and (**c**) 1.5 Ms.

Figure 3 illustrates the compressive strengths of the AASS and AASF mortars with various Ms values. The compressive strengths of all the specimens increased as time elapses. The compressive strength of GS1 developed rapidly at an early age but slowly at the later age (Figure 3a). There was more Ca and less Al in the slag (Table 1). The Al-O-Si bond is stronger than the Ca-O-Si bond [30]. The early reaction of GS1 was intense due to the faster dissolution of slag. Moreover, the shape of the slag was irregular (Figure 2a), which is conducive to the interlocking structure and increasing internal friction. However, the hydration products were wrapped around the unreacted slag particles. Hence, the later compressive strength growth was slower. The compressive strength of AAS mortar was markedly reduced by adding FA. This is related to the low activity and dilution effect of FA [31]. In particular, hollow spherical FA particles cause stress concentration [30]. Adding SS decreases the compressive strength of AAS mortar because of its low activity [32,33]. The reaction rate of SS was lower than that of slag. The f-CaO in SS leads to expansion that may produce small pores [34], resulting in a lower compressive strength. The compressive strength of SS1 was lower than that of GS1 but higher than that of FA1. This is attributed to the fast dissolution rate of SS at low Ms resulting in a high reaction rate and large amounts of products. The early compressive strength of GS2 grew faster (Figure 3b). The 3 d strength reached 62.0% of the 90 d strength. FA2 had a slightly lower compressive strength compared with SS2 at 3 d, which is significantly different from the result at 0.5 Ms (Figure 3a). A contrary finding was found after 3 d. The reaction rate decreased because of the slow dissolution rate of the SS particles at high Ms. The inert RO phase in SS has poor connectivity with the surrounding hydrates. However, the dissolution rate of FA is high at high Ms. FA particles reacted faster to generate C-A-S-H gel and improve the strength. The compressive strength of GS3 also grew faster at the early stage (Figure 3c). The 3 d strength reached 78.5% of the 90 d strength. The higher the Ms was, the more violent the reaction of the slag. The addition of SS or FA still reduced the strength of AAS mortar at 1.5 Ms, where the strength of SS3 was reduced more obviously. This is mainly related to the low dissolution rate of SS at high Ms. The CH crystals formed by the SS reaction tend to form weak links in the matrix, which are detrimental to strength development [31].

Figure 3a–c shows that the compressive strengths of the AAS and AASF mortars grew substantially with the increase in Ms. The compressive strengths of GS1 were only 54.1% and 73.0% of the compressive strengths of GS3 at 3 d and 90 d, respectively. The strengths of FA1 were only 41.5% and 54.2% of the strengths of FA3 at 3 d and 90 d, respectively. More silicate ions were provided, and more Si-O-Si bonds were formed at high Ms. The bonding energy of Si-O-Si was strong compared to that of Si-O-Al and Al-O-Al, which is conducive to an increase in strength [32,33]. In addition, the increase in Ms allowed for the rapid dissolution of Ca^2+^, Al^3+^, and Si^4+^ ions from the slag to participate in the reaction [35]. A larger amount of initial C-A-S-H gels were found at high Ms, which promotes the formation of denser microstructures [36,37]. The Ms had a major effect on early compressive strength of the AAS and AASF mortars but had a smaller influence on the later strength. For the AASS mortars, there was a little difference in the compressive strengths between SS1 and SS2. However, the compressive strength of SS3 was significantly increased at 3 d, while the compressive strength of SS1 at 90 d was 90.2% and 78.0% of that of SS2 and SS3, respectively. This is attributed to the dissolution effect of Ms on raw materials and the difference in activity between SS and slag. The Ms has a smaller effect on the early and later compressive strengths of the AASS mortars.

**Figure 4:**
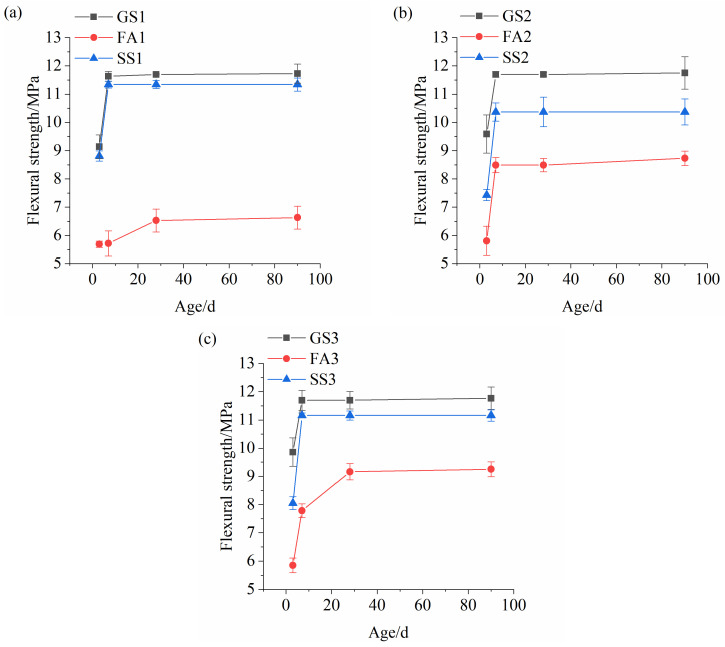
Flexural strengths of AASS and AASF mortars with different Ms values: (**a**) 0.5 Ms, (**b**) 1.0 Ms and (**c**) 1.5 Ms.

Figure 4 exhibits the flexural strengths of the AASS and AASF mortars with different Ms values. The flexural strengths of all the samples increase as time elapses. The flexural strength of GS1 grew rapidly within 7 d and slowly after 7 d (Figure 4a). The reaction of slag accelerated under alkaline conditions at the early stage. The flexural strength of FA1 was significantly lower than that of GS1, and it grew slowly as time elapsed. This is related to the low activity and slow reaction of FA. The flexural strength of SS1 developed similarly to that of GS1, but it was slightly lower than that of GS1. This further confirms that the mechanical properties of the AASS mortar are better than those of the AASF mortar at low Ms. The flexural strength of FA2 was low compared to GS2, but the disparity in flexural strength between the two samples was significantly reduced (Figure 4b). The flexural strength of SS2 was significantly smaller than that of GS2, which is attributed to the low dissolution rate of SS at high Ms. The change trends of the flexural strengths of the samples at 1.5 Ms are similar to those at 1.0 Ms (Figure 4c). When Ms was low, the excitation effect of water glass with 0.5 Ms on FA was poor, resulting in a low activity of fly ash and slow reaction. Thus, the flexural strength of AASF mortar at 0.5 Ms was significantly lower than that of AASF mortar at 1.0 Ms and 1.5 Ms (Figure 4a–c). However, when Ms was high, the flexural strength of the AASS mortar was still larger than that of the AASF mortar. The reducing effect of FA on the flexural strength is more significant than that of SS.

The comparison of Figure 4a–c displays that the early flexural strengths of the AAS and AASF mortars increase with increasing Ms. The later strengths of the AAS mortars with different Ms values show little difference. However, the later flexural strength of the AASF mortar dramatically increases at high Ms. It is indicated that the water glass with high Ms significantly stimulates the activity of FA. The flexural strength of the AASS mortar first decreases and then increases with increasing Ms. This is the result of competitive dissolution between the slag and SS caused by the increase in Ms. Increasing Ms enhances the activation effect of the activator on slag and FA, while it is unfavorable for SS.

The flexural-strength-to-compressive-strength ratio is a vital indicator reflecting the toughness of a material. A high ratio implies high toughness and low brittleness [38,39]. The flexural-strength-to-compressive-strength ratios of the three materials were greater at low Ms than those at high Ms (Table 4), indicating that increasing Ms reduces the toughness of the material. Adding FA decreased the ratio of the AAS mortar. However, the opposite result was obtained after adding SS. This indicates that adding SS markedly increased the toughness of the AAS mortar.

### 3.2. Non-Evaporable Water Content

**Figure 5:**
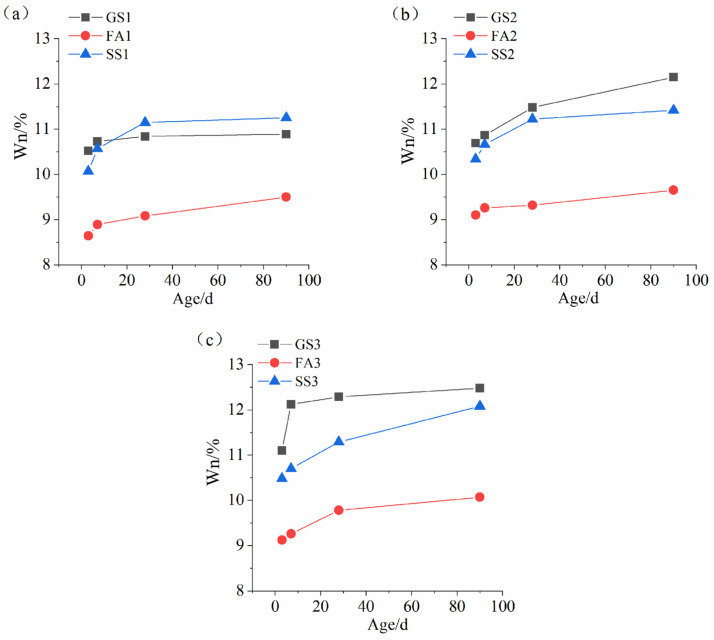
W_n_ of AASS and AASF pastes with different Ms values: (**a**) 0.5 Ms, (**b**) 1.0 Ms and (**c**) 1.5 Ms.

Figure 5 illustrates the W_n_ of the AASS and AASF pastes with different Ms values. The W_n_ increases with time for all specimens, revealing that the number of hydration products increases as the reaction proceeds. The W_n_ of GS1 had a high growth rate at the early stage and soon levelled off (Figure 5a). The addition of FA greatly reduced the W_n_ of the AAS paste, which is consistent with the strength results (Figure 3a). SS reduced the early age W_n_ of the AAS paste. However, the W_n_ of SS1 exceeded that of GS1 at a later age. The W_n_ of SS1 was already significantly greater than that of GS1 at 28 d and 90 d. SS contains C_2_S and C_3_S, and the reaction of SS produced CH, which further stimulated the reaction of slag. Meanwhile, the reaction rate of SS was higher at low Ms. As a consequence, the higher later-age W_n_ of SS1 was obtained. When Ms value increased to 1.0, the W_n_ of SS2 and FA2 were both smaller than that of GS2, but the W_n_ of SS2 was higher than that of FA2. The replacement of slag with SS or FA reduced the reaction degree of the AAS binder at 1.0 Ms, and the reduction was more pronounced after adding FA. Slag had the highest activity, and the reaction of slag produced the most hydration products. The CaO content of SS was higher than that of FA (Table 1). A large amount of Ca^2+^ made the SS react and produce abundant C-S-H gel and CH. Hence, the W_n_ of SS2 was higher than that of FA2. The variation pattern of the W_n_ at 1.5 Ms was consistent with that at 1.0 Ms (Figure 5c). However, the early W_n_ of GS3 improved remarkably, and it was much higher than that of SS3 and FA3. This alludes to the fact that the early reaction degree of slag was distinctly high at 1.5 Ms. Water glass with high Ms provides more silicate ions, which promotes the reaction. The higher growth rate of the early W_n_ of FA3 implies that increasing Ms accelerates the early reaction of the AASF binder. The incorporation of SS significantly decreased the W_n_ of the AAS paste at 1.5 Ms. However, the gap between the W_n_ of SS3 and that of GS3 evidently shortened at 90 d. The dissolution rate of SS was reduced at high Ms, resulting in a low early reaction rate. However, the increase in silicate ions and the mutual promotion reaction effect of SS and slag caused the high growth rate of the later W_n_.

The comparison of Figure 5a–c shows that an increase in Ms increased the W_n_ of specimens, implying that increasing Ms promotes the reaction of the AASS and AASF binders. Increasing Ms can not only promote the rapid dissolution of slag but also directly participates in the polymerization reaction as a reactant, thus increasing the amount of hydration products. The early growth rate in the W_n_ of the AASS paste decreased with increasing Ms, indicating that the reaction degree of SS was low when Ms was high. Note that the development patterns of the W_n_ and compressive strengths of AASS and AASF binders are not consistent. The W_n_ of the AASS paste was sharply higher than that of the AASF paste at high Ms, but the compressive strength was the opposite. This is related to the various types of hydration products. The formation of CH due to SS reaction was not conducive to the development of strength. In addition, the RO phase does not tightly bond with the surrounding hydrates, which ultimately leads to low compressive strength.

### 3.3. XRD

**Figure 6:**
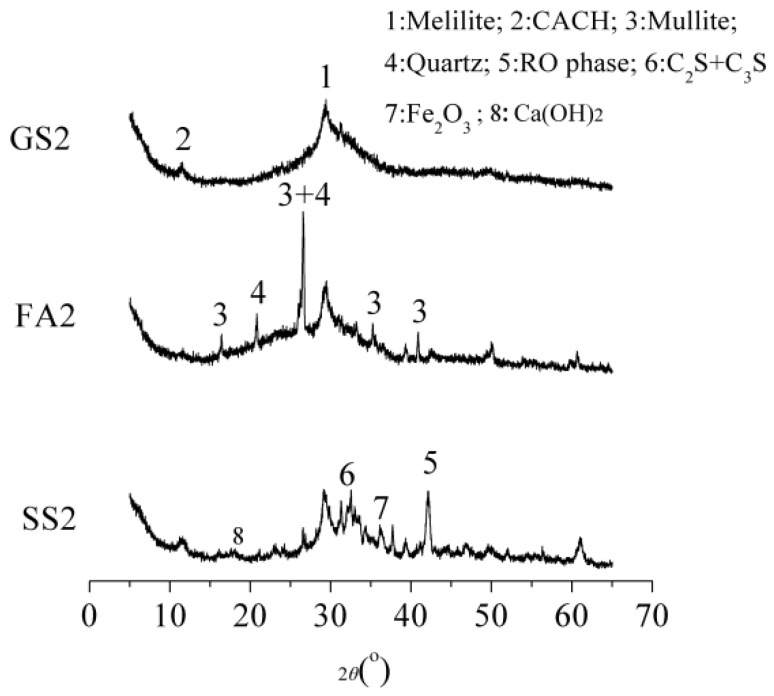
The XRD patterns of the AASS and AASF pastes at 28 d and 1.0 Ms.

Figure 6 exhibits the XRD patterns of the AASS and AASF pastes at 28 d and 1.0 Ms. The intensities of the diffraction peaks of melilite and the calcium carboaluminate hydrate (CACH) phase in GS2 are strong. Melilite and CACH are the mineral composition and the hydration product of slag, respectively. This indicates that the slag in GS2 reacts and forms CACH at 28 d. Melilite minerals were still present in the specimen, confirming that the slag was not completely reacted at 28 d. The diffraction peaks of quartz and mullite in FA2 are more obvious, and the intensities of the diffraction peaks are strong. These two phases are the mineral compositions of FA, illustrating that FA did not completely reacted. The intensity of the diffraction peak of melilite in FA2 was slightly lower than that in GS2, inferring that the amount of unreacted slag in FA2 is still large. The intensities of the diffraction peaks of C_2_S, C_3_S, RO phase, and Fe_2_O_3_ in SS2 were strong. The reaction rates of C_2_S and C_3_S were slow. The inert RO phase and Fe_2_O_3_ phase in SS2 did not participate in the reaction. The unreacted SS still existed at 28 d. The intensity of the diffraction peak of melilite in SS2 was dramatically weaker than that in GS2. This elucidates that the reaction degree of slag in SS2 was considerably greater. The diffraction peak of CH in SS2 was also found. The mutual promotion between slag and SS in the reaction process improved the reaction degrees of slag and SS.

### 3.4. Pore Structure Analysis

**Figure 7:**
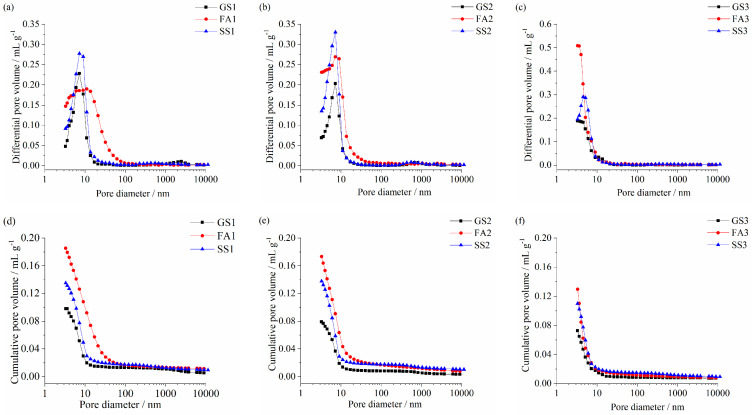
Differential pore volume: (**a**) 0.5 Ms, (**b**) 1.0 Ms and (**c**) 1.5 Ms and cumulative pore volume (**d**) 0.5 Ms, (**e**) 1.0 Ms and (**f**) 1.5 Ms of the AASS and AASF pastes at 90 d.

Figure 7 shows the differential pore volume and cumulative pore volume of the AASS and AASF pastes at 90 d. The most probable pore diameters of GS1, FA1, and SS1 are 8 nm, 13 nm, and 8 nm, respectively (Figure 7a). As explained above, the lower activity of FA results in a larger diameter being the most probable pore. The most probable pore diameter was almost unchanged after incorporating SS. However, the cumulative pore volume was increased (Figure 7d). A smaller number of hydration products were formed in the AASS paste compared with the AAS paste at 90 d (Figure 5b). In addition, the larger particle size of the RO phase in SS also made it difficult to fill the pores. Therefore, the cumulative pore volume was larger. The cumulative pore volume of SS1 was lower than that of FA1 (Figure 7d). The most probable pore diameters of all the specimens decreased to 7.5 nm at 1.0 Ms (Figure 7b). The cumulative pore volume of SS2 was still lower than that of FA2. When Ms increased to 1.5, the most probable pore diameters of GS3, FA3, and SS3 pastes decreased to 3.5 nm, 3.5 nm, and 5 nm, respectively (Figure 7c). The pore structure was further refined. More hydration products were generated at 1.5 Ms (Figure 5c), the microstructure was denser, and the compressive strength was higher (Figure 3c). The cumulative pore volume increased when SS or FA was added. Adding FA raised the cumulative pore volume even more (Figure 7f). With the increase in Ms, the cumulative pore volume of three specimens decreased significantly. Notably, the cumulative pore volumes of the AASS paste were smaller than those of AASF paste at 1.0 and 1.5 Ms, but the results of the compressive strength were reversed. This is because SS2 and SS3 have larger pore volumes (>100 nm), which is harmful to their mechanical properties. Meanwhile, the hydration product CH of SS is not conducive to compressive strength.

**Figure 8:**
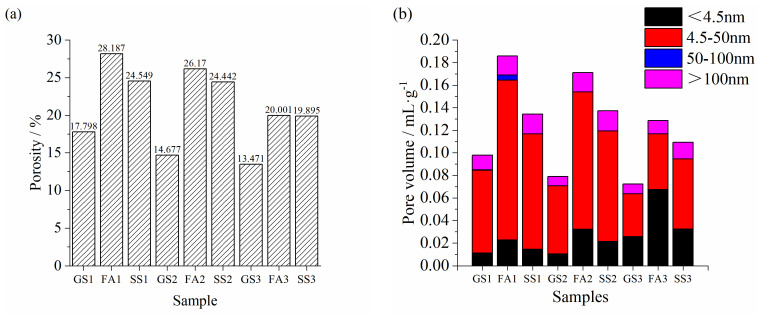
The porosity and pore volume of the AASS and AASF pastes: (**a**) porosity and (**b**) pore volume.

Figure 8a,b exhibits the porosity and pore volume of the AASS and AASF pastes at 90 d, respectively. The incorporation of SS or FA increased the porosity of the AAS paste at the three studied Ms, and the porosity of FA paste was significantly high (Figure 8a). The porosity of all the samples decreased with increasing Ms. The pore structure of the paste mostly depended on the amount of hydrates. More hydration products resulted in a denser microstructure. The AAS paste had the most abundant hydration products, followed by the AASS paste, and the AASF paste had the lowest content of hydration products (Figure 5). When slag was partly replaced by SS or FA, the amount of slag was relatively reduced. The decreased reaction degree led to the formation of fewer hydration products. The pores were not adequately filled. Hence, the porosity was high. The compressive strength was also decreased (Figure 3).

The pore size distribution was divided into four ranges: harmless pores (<4.5 nm); small harmful pores (4.5–50 nm); harmful pores (50–100 nm); and multiple harmful pores (>100 nm). The harmful and multiple harmful pores are the main focus of this research. The small harmful and harmless pores have little impact on the mechanical properties of the paste. GS1 had the least multiple harmful and harmful pores, while FA1 had the most harmful and multiple harmful pores (Figure 8b). As a result, FA1 had the lowest compressive strength (Figure 3a). When the Ms values were 1.0 and 1.5, the addition of SS made this paste have the most harmful and multiple harmful pores. Therefore, the compressive strengths of SS2 and SS3 were the lowest (Figure 3b,c). The volumes of multiple harmful and harmful pores in the AAS and AASF pastes obviously decreased with increasing Ms, while the volume of harmless pores increased. An increasingly dense structure and higher strength were obtained. For the AASS paste, the volume of multiple harmful pores and the cumulative pore volume of SS1 and SS2 showed little difference. Therefore, the difference in compressive strength between SS1 and SS2 was not significant at 90 d. (Figure 3a,b). However, the volume of harmless pores in SS3 increased, and the volume of multiple harmful pores decreased significantly. Furthermore, the porosity also evidently decreased, which is in line with the change trend in compressive strength (Figure 3). The alkali content of the activator was the same. When the silicate modulus increased from 0.5 to 1.5, the concentration of SiO_2_ increased. It could promote the reaction of binders, increase the reaction degree, and improve the pore structure of paste.

### 3.5. Morphology

**Figure 9:**
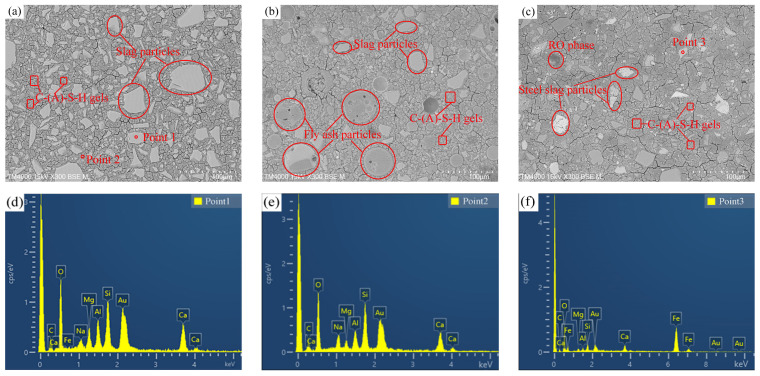
BSE images and EDS results of AASS and AASF pastes at 28 d and 1.0 Ms: (**a**) GS2; (**b**) FA2; (**c**) SS2; (**d**) point 1; (**e**) point 2; (**f**) point 3.

Figure 9 shows the BSE images and EDS results of the AASS and AASF pastes at 28 d and 1.0 Ms. Unreacted slag particles were clearly observed in GS2 (Figure 9a). The particle marked as point 1 confirms slag with high contents of Mg (Figure 9d). The hydration products closely surrounded the unreacted slag particles. The microstructure was much denser. Thus, the porosity was the lowest and the strength was the highest (Figure 3b and Figure 8a). The EDS result of point 2 is presented in Figure 9e. The hydration product was C(N)-(A)-S-H gel, which contains elemental Na because of the use of water glass as an activator. This is consistent with the XRD results (Figure 6). Due to the large shrinkage of the AAS paste, shrinkage cracks can be observed in the BSE images. The reactions of the FA and slag occurred under the activation of water glass (Figure 9b). The addition of FA significantly reduced the cracks. There were still many unreacted FA particles at 28 d. The microstructure was looser compared to that of GS2. The number of hydration products generated by the reaction was smaller (Figure 5b). Hence, the structure was relatively loose. As a result, the strength of FA2 was lower (Figure 3). Unreacted SS and slag particles were found in SS2 (Figure 9c). The connections between the substrates were not tight enough. The structure of SS2 was looser than that of FA2. Thus, the strength of SS2 was lower than that of FA2 (Figure 3b). A larger relative atomic number in the phase results in a larger grayscale and brighter brightness. The EDS result of point 3 shows a stronger peak intensity for elemental Fe (Figure 9f). Bright gray is also found. These results confirm that it is an unreacted SS particle. The darker colored area is the RO phase, which does not participate in the reaction.

### 3.6. Analysis of the Ca/Si Ratio and Al/Si Ratio

**Figure 10:**
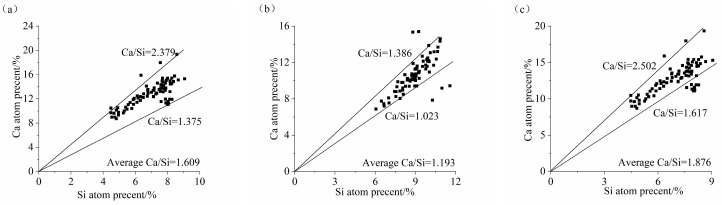
Ca/Si ratio of C-(A)-S-H gel of AASS and AASF pastes at 1.0 Ms: (**a**) GS2, (**b**) FA2 and (**c**) SS2.

**Figure 11:**
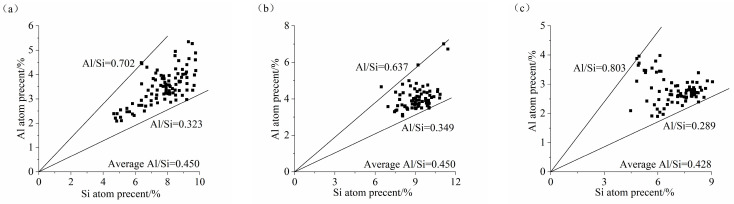
Al/Si ratio of C-(A)-S-H gel of AASS and AASF pastes at 1.0 Ms: (**a**) GS2, (**b**) FA2 and (**c**) SS2.

The main hydration product of the AASS and AASF binders was C-(A)-S-H gel. Their properties directly affect the performance of concrete. Figure 10 and Figure 11 show the Ca/Si and Al/Si ratios of the C-(A)-S-H gel of the AASS and AASF pastes at 28 d and 1.0 Ms, respectively. The Ca/Si ratios of the C-(A)-S-H gel of GS2, FA2, and SS2 were 1.375~2.379, 1.023~1.386, and 1.617~2.502, respectively, and their average Ca/Si ratios were 1.609, 1.193, and 1.876, respectively. The Al/Si ratios of the C-(A)-S-H gel of GS2, FA2, and SS2 were 0.323~0.702, 0.349~0.637, and 0.289~0.803, respectively, and their average Al/Si ratios were 0.450, 0.450, and 0.428, respectively. Adding FA reduced the Ca/Si ratio of C-(A)-S-H gel. FA had a lower CaO content and a higher SiO_2_ content than slag (Table 1). The replacement of slag with FA reduced the Ca^2+^ concentration. Therefore, the incorporation of FA reduced the Ca/Si ratio of the C-(A)-S-H gel. Although the contents of Al and Si in FA were higher than those in slag, the ratio of Al and Si atoms in the two raw materials was not much different. As a consequence, the difference in the Al/Si ratio between GS2 and FA2 was not significant. The addition of SS increased the Ca/Si ratio of the C-(A)-S-H gel. SS had a higher CaO content and lower SiO_2_ content than slag (Table 1). The substitution of slag with SS increased the Ca^2+^ concentration and reduced the Si^4+^ concentration. However, the hydration degrees of C_3_S and C_2_S in SS were low due to the low activity of SS. Thus, the addition of SS slightly increased the Ca/Si ratio of the C-(A)-S-H gel in the AAS paste. However, the Al/Si ratio was reduced by adding steel slag, as a result of too little Al in the SS (Table 1). Currently, there is no consensus regarding the influence of Ca/Si and Al/Si ratios on the mechanical properties because of the different types and amounts of hydration products generated. Commonly, a lower Ca/Si ratio is better for these properties. The incorporation of Al in the C-S-H gel improved the properties of the material, and a higher Al/Si ratio might be better for properties.

### 3.7. Drying Shrinkage

**Figure 12:**
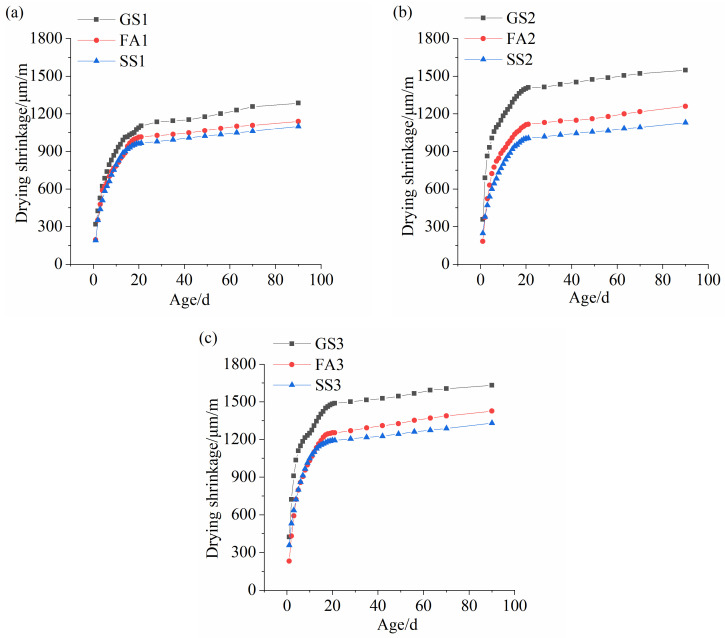
Drying shrinkages of AASS and AASF mortars with different Ms values: (**a**) 0.5 Ms, (**b**) 1.0 Ms and (**c**) 1.5 Ms.

Figure 12 shows the drying shrinkages of the AASS and AASF mortars with different Ms values. The drying shrinkage of the AAS mortar was the largest, followed by that of the AASF mortar, and the drying shrinkage of the AASS mortar was the smallest. This is in accordance with the morphology results (Figure 9). The drying shrinkage mainly occurred in the first 21 d. The drying shrinkage resulted from the evaporation of water in the capillaries [40]. The smaller the capillaries were, the higher the capillary pressure when water evaporated. The principal factors affecting the drying shrinkage of AAMs are precursor, activator type, silicate modulus, curing conditions, and hydration product type [14,41,42,43,44]. It is clear that the drying shrinkage of the AAS mortar was significantly reduced by adding SS or FA at all the studied Ms. Additionally, the reduction effect of SS on drying shrinkage was more significant.

The rapid reaction of slag produces C-(A)-S-H gel. The addition of the basic metal cation Na^+^ to C-(A)-S-H gels reduced the regular arrangement of C-(A)-S-H gels, making them susceptible to collapse and rearrangement during drying [45,46]. Therefore, a larger drying shrinkage was obtained. The lower activity of FA led to the formation of smaller amounts of hydration products (Figure 5). Simultaneously, FA had a microaggregate effect and diminished drying shrinkage [47]. The microaggregate effect of SS was also obvious because of the high hardness and fine particles. Apart from the C-(A)-S-H gels, there were CH crystals in the hydration products of SS, which inhibited the shrinkage of the system. The Ca/Si ratio of C-(A)-S-H gels increased when adding SS (Figure 10). A higher Ca/Si ratio leads to lower shrinkage [48]. The microexpansion effect produced by the reaction of f-CaO with water in the SS can also compensate for the volume shrinkage [49].

The drying shrinkages of all the samples increased with increasing Ms. An increase in Ms decreased the pore size (Figure 7), enhanced the densification degree of the pore structure, and increased the pore pressure [50]. Therefore, the drying shrinkage increased. In addition, the water glass with higher Ms presented greater drying shrinkage [51].

## 4. Conclusions

Many researchers commonly use FA to reduce the cost and drying shrinkage of AAS binder and to regulate the mechanical properties of AAS binder. By comparing the effects of SS and FA on the strength, microstructure, and drying shrinkage of AAS binder, the feasibility of using SS to improve the performance of AAS paste was studied. The specific conclusions are as follows:

(1) Adding SS or FA reduced the mechanical properties of the AAS mortar. SS significantly reduced the compressive strength of AAS mortar at high Ms, but FA evidently decreased the compressive strength of AAS mortar at low Ms. The toughness of AASS mortar was better than that of AASF mortar. The compressive strengths of all the samples increased with increasing Ms.
(2) The incorporation of SS increased the later-age W_n_ of the AAS paste at low Ms but reduced the W_n_ at high Ms. The addition of FA markedly reduced the W_n_ of AAS paste. The W_n_ of AASS paste was notably high compared to that of AASF paste.
(3) The addition of SS or FA increased the cumulative pore volume and porosity of the AAS paste, especially after adding FA. Increasing the Ms refined the pore structures of all the samples.
(4) The AAS paste becomes looser after adding SS or FA. The C-(A)-S-H gels and unreacted SS or FA particles were observed. The microstructure became denser with increasing Ms.
(5) Adding SS increased the Ca/Si ratio of the C-(A)-S-H gel but decreased the Al/Si ratio. However, the Ca/Si ratio was reduced, and the Al/Si ratio was almost unchanged by adding FA.
(6) Adding SS or FA markedly reduced the drying shrinkage of the AAS mortar at all the studied Ms. The reduction effect of SS on drying shrinkage was more significant.
(7) SS and slag both belong to the iron and steel industry. SS can replace FA to prepare AAS composite binder.

## Figures and Tables

**Table 1:** Chemical compositions of slag, FA, and SS (wt/%).

Chemical Compositions	SiO_2_	Al_2_O_3_	Fe_2_O_3_	CaO	MgO	SO_3_	Na_2_O_eq_	LOI	Others
Slag	35.55	15.36	0.45	33.94	11.16	1.95	0.63	0.70	0.96
FA	57.60	21.90	7.70	3.87	1.68	0.41	4.05	0.43	2.79
SS	12.77	2.12	23.49	49.17	3.54	0.23	0.45	1.86	8.23

Na_2_O_eq_ = Na_2_O + 0.658K_2_O. LOI: Loss on ignition.

**Table 2:** Mix proportions of the pastes (wt/%).

Sample	Slag	SS	FA	Ms
GS1	100	0	0	0.5
SS1	50	50	0	
FA1	50	0	50	
GS2	100	0	0	1.0
SS2	50	50	0	
FA2	50	0	50	
GS3	100	0	0	1.5
SS3	50	50	0	
FA3	50	0	50	

**Table 3:** Mix proportions of the mortars.

Sample	Slag (g)	SS (g)	FA (g)	Standard Sand (g)	Water (g)	Ms
GS1	450	0	0	1350	154.14	0.5
SS1	225	225	0			
FA1	225	0	225			
GS2	450	0	0	1350	133.50	1.0
SS2	225	225	0			
FA2	225	0	225			
GS3	450	0	0	1350	112.86	1.5
SS3	225	225	0			
FA3	225	0	225			

**Table 4:** Flexural-strength-to-compressive-strength ratios of specimens at different ages (%).

Age/d	GS1	SS1	FA1	GS2	SS2	FA2	GS3	SS3	FA3
3	23.0	25.6	26.7	17.7	19.9	16.0	13.4	15.9	11.0
7	24.3	30.4	23.7	19.0	23.8	17.8	14.1	19.0	12.1
28	19.6	23.1	17.8	15.3	20.4	14.7	13.9	16.5	12.0
90	17.1	15.2	20.5	13.4	13.4	16.9	12.6	11.5	15.8

## Data Availability

Data are contained within the article.
